# Bupivacaine suppresses the progression of gastric cancer through regulating circ_0000376/miR-145-5p axis

**DOI:** 10.1186/s12871-020-01179-4

**Published:** 2020-10-30

**Authors:** Changqiao Ju, Jia Zhou, Hui Miao, Xin Chen, Qingyu Zhang

**Affiliations:** 1 Department of Anesthesiology, The Second Affiliated Hospital of Mudanjiang, No. 51 Kangjia Street, Aimin District, Mudanjiang City, 157011 Heilongjiang Province China; 2 Department of Ultrasound, The Second Affiliated Hospital of Mudanjiang, Mudanjiang, Heilongjiang China; 3 Department of Gynaecology and Obstetrics, The Second Affiliated Hospital of Mudanjiang, Mudanjiang, Heilongjiang China

**Keywords:** Gastric cancer, Bupivacaine, circ_0000376, miR-145-5p, Progression, Glycolysis

## Abstract

**Background:**

Local anesthetic Bupivacaine commonly used in gastric cancer resection operation has been reported to suppress the progression of gastric cancer. However, the specific mechanism by which Bupivacaine functions is largely unexplored.

**Methods:**

The viability and metastasis of gastric cancer cells were assessed by Cell counting kit-8 (CCK8) assay and transwell migration and invasion assays. The apoptosis was evaluated by caspase-3 activity detection assay and flow cytometry. The glycolysis was analyzed through detecting the extracellular acidification rate (ECAR) via Seahorse XF 96 Extracellular Flux Analyzer and the expression of glucose transporter type 1 (GLUT1) and lactic dehydrogenase A (LDHA) via Western blot assay. Quantitative real-time polymerase chain reaction (qRT-PCR) was applied to detect the expression of circular RNA 0000376 (circ_0000376) and microRNA-145-5p (miR-145-5p). The interaction between circ_0000376 and miR-145-5p was predicted using Circular RNA Interactome database and validated by dual-luciferase reporter assay.

**Results:**

Bupivacaine restrained the viability, metastasis and glycolytic process while promoted the apoptosis of gastric cancer cells. Bupivacaine decreased the level of circ_0000376 while enhanced the abundance of miR-145-5p in gastric cancer cells. Circ_0000376 accelerated the malignant behaviors of gastric cancer cells. MiR-145-5p directly interacted with circ_0000376 in gastric cancer cells, and miR-145-5p was negatively regulated by circ_0000376. The addition of circ_0000376 or the interference of miR-145-5p partly reversed Bupivacaine-mediated influences in gastric cancer cells.

**Conclusion:**

Bupivacaine exerted an anti-tumor role to suppress the progression of gastric cancer through reducing the abundance of circ_0000376 and up-regulating miR-145-5p.

## Background

Gastric cancer is a common malignancy globally [1]. Surgical resection and the eradication of *Helicobacter pylori* are the important treatment methods for gastric cancer [2, 3]. Nevertheless, the prognosis of gastric cancer patients at advanced stage remains dismal due to the metastasis and recurrence. Therefore, finding novel biomarkers with high specificity and high sensitivity is indispensable for the early diagnosis of gastric cancer.

Accumulating articles have pointed out that the type of anesthetic used in surgery might affect the long-term outcomes of diseases [4, 5]. Clinical researches also reported that local anesthesia suppressed the recurrence and motility of cancer patients who had accepted surgical resection [6, 7]. Bupivacaine is a local anesthetic commonly used in the resection operation of gastric cancer patients [8]. A large amount of articles suggested that Bupivacaine triggered the death and suppressed the proliferation of multiple cancer cells [9, 10]. Nevertheless, the underlying working mechanisms behind Bupivacaine remain to be elucidated.

Dysregulated non-coding RNAs (ncRNAs) were implicated in the initiation and progression of gastric cancer [11, 12]. Among these ncRNAs, circular RNAs (circRNAs) are ideal biomarkers for human diseases due to their covalently closed loop structure [13]. Li et al. claimed that circ_0008035 accelerated the progression of gastric cancer through regulating microRNA-599 (miR-599)/EIF4A1 axis [14]. CircPIP5K1A contributed to the development of gastric cancer through targeting miR-376c-3p/ZNF146 signaling [15]. Circ_0000376 has been reported to be significantly up-regulated in gastric cancer [16]. Here, we intended to uncover the role of circ_0000376 and illustrate the association between circ_0000376 and Bupivacaine in gastric cancer cells.

MicroRNAs (miRNAs) are regarded as a class of short ncRNAs with 18–24 nucleotides [17]. The abnormal expression of miRNAs was associated with the pathogenesis of multiple cancers. For instance, miR-520f-3p restrained cell proliferation of gastric cancer cells through regulating SOX9/Wnt signal pathway [18]. MiR-1271-5p suppressed the proliferation and elevated the radiotherapy sensitivity via CDK1 in hepatocellular carcinoma [19]. MiR-145-5p served as the downstream gene of circDUSP16 to suppress the progression of gastric cancer [20]. However, the potential mechanism of miR-145-5p in gastric cancer is still elusive.

Bupivacaine was found to suppress the viability, metastasis and glycolysis and promote the apoptosis of gastric cancer cells. Circ_0000376 and miR-145-5p levels could be regulated by Bupivacaine, and circ_0000376/miR-145-5p axis was identified to illustrate the molecular working mechanism of Bupivacaine in the progression of gastric cancer.

## Methods

### Cell culture

Human gastric cancer cell lines (AGS and HGC27) purchased from BeNa Culture Collection (Beijing, China) were used to conduct cellular experiments. HGC27 cell line was cultivated using Roswell Park Memorial Institute-1640 (RPMI-1640) medium (Gibco, Carlsbad, CA, USA), while Dulbecco’s modified Eagle’s medium/Nutrient Mixture F-12 (DMEM/F12; Gibco) was used for the cultivation of AGS cell line. 10% fetal bovine serum (FBS; Gibco) and 10% 100 units/mL penicillin/100 μg/mL streptomycin mixture were added to complete the base medium. These cell lines were maintained in an 37 °C incubator containing 5% CO_2_.

### Bupivacaine

Bupivacaine was purchased from Sigma (St. Louis, MO, USA), and it was used at the final concentration of 1 μg/mL, 5 μg/mL or 10 μg/mL to simulate clinical condition.

### Cell counting kit-8 (CCK8) assay

Gastric cancer cells were plated in the 96-well plates at the concentration of 3 × 10^3^ cells per well in sextuplicate. The next day, transfection or drug treatment was conducted, and 10 μL CCK8 reagent (Dojindo, Tokyo, Japan) was added to the culture medium to incubate with the transfected gastric cancer cells. Cells were continued to cultivate for 1 h in normal condition in the cell culture incubator, and the cell viability was examined through determining the optical density at 450 nm. The experiment was repeated for three times.

### Caspase-3 activity detection assay

A commercial caspase-3 activity detection colorimetric assay kit (KeyGen, Jiangsu, China) was used in this experiment. Gastric cancer cells were disrupted and divided into two equal parts. The protein samples were mixed with 2 × reaction buffer and substrate at room temperature in a dark room for 4 h. The optical density was measured at 405 nm. The experiment was repeated for three times.

### Flow cytometry

Gastric cancer cells were seeded in the 6-well plates at suitable concentration. The next day, Bupivacaine treatment or transfection was performed. The treated gastric cancer cells were rinsed using phosphate buffered saline (PBS) and re-suspended in binding buffer. Subsequently, 5 μL fluorescein isothiocynate (FITC)-conjugated Annexin V (Solarbio, Beijing, China) and 5 μL propidium iodide (PI; Solarbio) were simultaneously added to the tubes to incubate with the cells in a dark room. Finally, the apoptotic gastric cancer cells (early stage and late stage) were identified from normal or necrotic cells by the flow cytometer. The experiment was repeated for three times.

### Transwell migration and invasion assays

Transwell chambers (Costar, Corning, NY, USA) along with Matrigel (BD biosciences, San Jose, CA, USA) or not were used to analyze the invasion or migration ability of gastric cancer cells, respectively. After pre-coating with Matrigel (invasion assay) or not (migration assay), 100 μL gastric cancer cell suspension (without serum) was added to the upper chambers at a concentration of 5 × 10^4^ (invasion assay) or 1 × 10^4^ cells (migration assay)/well. 500 μL 10% FBS-contained medium was added to the lower chambers to act as chemotactic factor. After 24-h incubation, migration and invasion gastric cancer cells were stained with 0.5% crystal violet (Sigma), and the cell number was counted at five random fields. The magnification in the images is 100. The experiment was repeated for three times.

### Extracellular acidification rate (ECAR) analysis

The Seahorse XF 96 Extracellular Flux Analyzer (Agilent Technologies, Santa Clara, CA, USA) and Seahorse XF Glycolysis Stress Test Kit (Agilent Technologies) were used to detect the ECAR. Gastric cancer cells were seeded into the wells of the Seahorse XF plate at a density of 1 × 10^4^ cells/well. 10 mM Glucose, 1 μM Oligomycin and 50 mM 2-deoxyglucose (2-DG) were sequentially added to the wells at the indicated time points. The cell number was analyzed through crystal violet staining. The glycolysis rate and glycolytic capacity were calculated as previously reported [21].

### Western blot assay

Protein samples were extracted using cell lysis buffer (Abcam, Cambridge, MA, USA) containing protease inhibitor. Protein samples (25 μg) were separated by sodium dodecyl sulfate polyacrylamide gel electrophoresis (SDS-PAGE) gel and transferred to the polyvinylidene difluoride (PVDF) membrane (Bio-Rad, Hercules, CA, USA). Subsequently, the membrane was blocked using 5% skimmed milk, and then incubated with specific primary antibodies overnight at 4 °C. The primary antibodies included anti-glucose transporter type 1 (anti-GLUT1; ab115730; Abcam), anti-lactic dehydrogenase A (anti-LDHA; ab226016; Abcam) and anti-β-actin (ab8227; Abcam). β-actin was used as the internal reference. The horseradish peroxidase (HRP)-conjugated secondary antibody was then incubated with the membrane for 2 h at room temperature. The protein signals were examined by the enhanced chemiluminescent visualization (ECL) system (Pierce Biotechnology, Rockford, IL, USA). The experiment was repeated for three times.

### Quantitative real-time polymerase chain reaction (qRT-PCR)

RNA isolation was conducted with Trizol solution (Invitrogen, Carlsbad, CA, USA). Template DNA of circ_0000376 and miR-145-5p was synthesized using the Bio-Rad iScript kit (Bio-Rad) and TaqMan reverse transcription kit (Applied Biosystems, Rotkreuz, Switzerland), respectively. PCR reaction of circ_0000376 and miR-145-5p was conducted with iQSYBR Green SuperMix (Bio-Rad) and TaqMan MicroRNA assay kit (Applied Biosystems), respectively. Glyceraldehyde-3-phosphate dehydrogenase (GAPDH) and U6 small nuclear RNA (snRNA) were used as the internal controls for circ_0000376 and miR-145-5p, respectively. The relative expression was analyzed using the 2^-ΔΔCt^ method. The primers used in this experiment were listed in Table 1. The experiment was repeated for three times.

**Table 1:** Primer sequences used in qRT-PCR assay

Gene name	Primer sequence
circ_0000376	TTTGGATGTGGAGGGGAATA (forward)
	GAGCCCAGGAGTTCCAGACT (reverse)
miR-145-5p	GUCCAGUUUUCCCAGGAAUCCCU (forward)
	AGGGATTCCTGGGAAAACTGGAC (reverse)
U6	GCGCGTCGTGAAGCGTTC (forward)
	GTGCAGGGTCCGAGGT (reverse)
GAPDH	GGTCGGAGTCAACGGATTTG (forward)
	ATGAGCCCCAGCCTTCTCCAT (reverse)

### Cell transfection

Gastric cancer cells were plated into 6-well plates at suitable concentration overnight. When cell confluence reached about 70%, circ_0000376 specific small interfering RNAs (si-circ_0000376#1, si-circ_0000376#2 and si-circ_0000376#3), siRNA negative control (si-NC), circ_0000376 overexpression vector (circ_0000376), pLCDH-cir (vector), miR-145-5p mimic (miR-145-5p), miR-NC, miR-145-5p inhibitor (anti-miR-145-5p) or anti-NC purchased from Sangon (Shanghai, China) and Genepharma (Shanghai, China) was transfected into gastric cancer cells with Lipofectamine™ 3000 (Thermo Fisher Scientific, Shanghai, China). The sequences of three siRNAs targeting circ_0000376 were listed as below.

Si-circ_0000376#1: 5′-AGAAUCCAACUCUCAUAUGGA-3′.

Si-circ_0000376#2: 5′-AAUCCAACUCUCAUAGGAUA-3′.

Si-circ_0000376#3: 5′-AUCCAACUCUCAUAUGGAUAG-3′.

### Bioinformatic prediction

Circular RNA Interactome database was used for seeking the direct downstream miRNA targets of circ_0000376 based on the complementary sites between circ_0000376 and miRNAs.

### Dual-luciferase reporter assay

The sequence of circ_0000376 containing putative complementary sites (ACUGGA) with miR-145-5p (UGACCU) was amplified and inserted into pGL3 plasmid (Ambion, Austin, TX, USA), named as circ_0000376 wild-type (wt). Site-directed mutation in miR-145-5p binding sites of circ_0000376 sequence was also created and cloned into pGL3 plasmid (Ambion), named as circ_0000376 mut. Gastric cancer cells were seeded into 24-well plates. The next day, 120 ng circ_0000376 wt or circ_0000376 mut and 40 nM miR-145-5p or miR-NC were co-transfected into gastric cancer cells. The Firefly and Renilla fluorescence intensities were detected by the Dual-luciferase reporter assay system (Promega, Madison, WI, USA). Firefly activity was normalized to Renilla activity. The experiment was repeated for three times.

### Statistical analysis

Data were displayed as mean ± standard deviation (SD) from at least three independent assays. The comparisons between two groups were conducted using Student’s *t*-test, while the differences among more than two groups were analyzed using one-way analysis of variance (ANOVA) followed by Tukey’s test. Statistical significance was defined when *P* value less than 0.05.

## Results

### Bupivacaine suppresses cell viability, migration and invasion while induces the apoptosis of gastric cancer cells

**Fig. 1:**
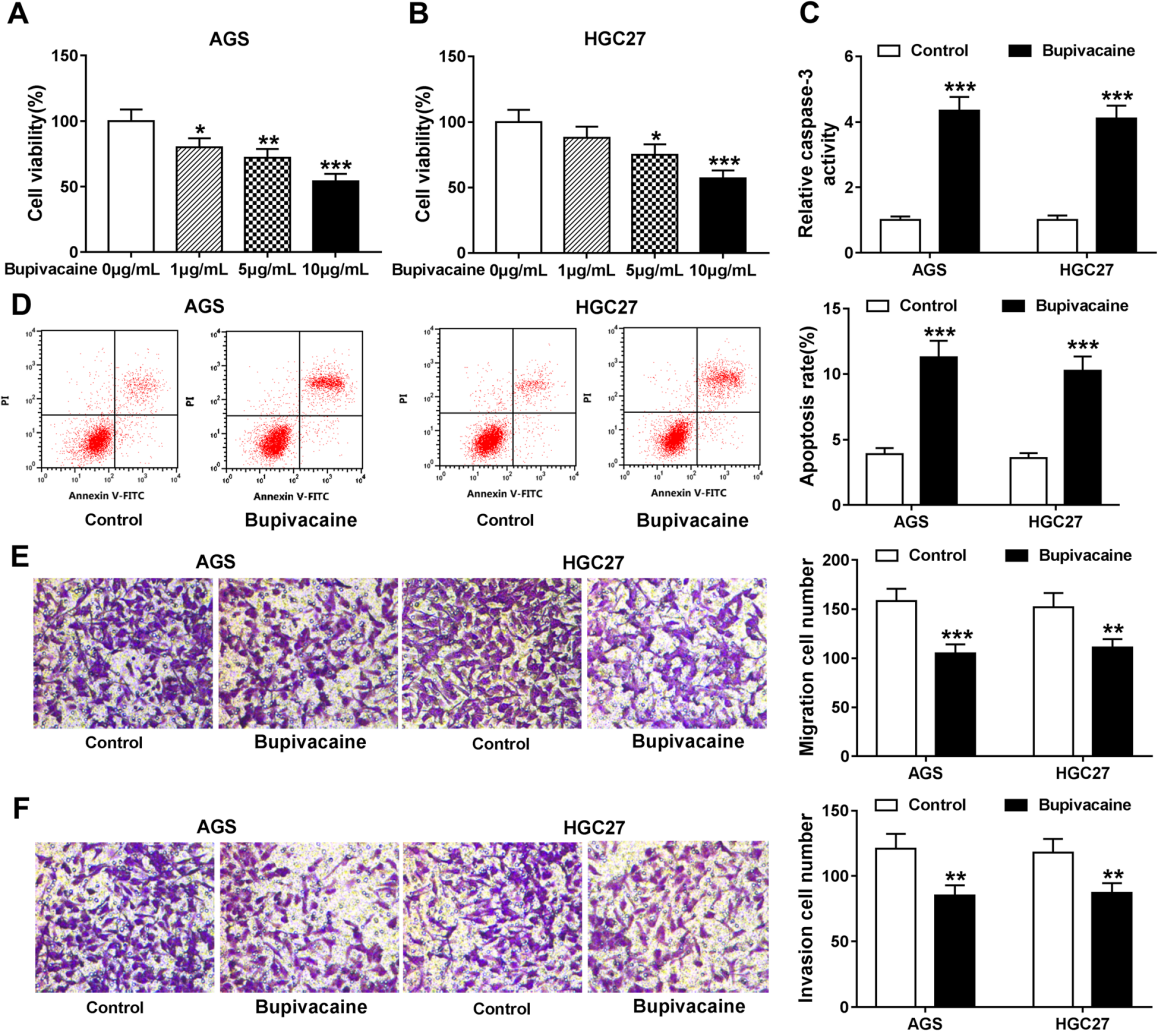
Bupivacaine suppresses cell viability, migration and invasion while induces the apoptosis of gastric cancer cells. **a** and **b** AGS and HGC27 cells were exposed to different doses of Bupivacaine (1 μg/mL, 5 μg/mL or 10 μg/mL). CCK8 assay was used to measure the viability of gastric cells after 48-h treatment. **c-f** AGS and HGC27 cells were treated with 10 μg/mL Bupivacaine for 48 h. **c** Caspase-3 activity in AGS and HGC27 cells was examined to analyze the apoptosis of gastric cells using Caspase-3 activity detection kit. **d** The apoptotic gastric cancer cells (early stage and late stage) were identified by flow cytometry. **e** and **f** Transwell migration and invasion assays were performed to assess the migration and invasion abilities of Bupivacaine-treated gastric cancer cells. The magnification in the images is 100. *P < 0.05, **P < 0.01, ***P < 0.001

To clarify the role of anesthetic Bupivacaine in the progression of gastric cancer, two gastric cancer cell lines (AGS and HGC27) were used to explore the functions of Bupivacaine in the viability, apoptosis, migration and invasion abilities of gastric cancer cells in vitro. After treating increased doses of Bupivacaine for 48 h, the number of viable cells was analyzed by CCK8 assay. Bupivacaine inhibited the viability of gastric cancer cells, and the viability was gradually reduced along with the increased treatment concentration of Bupivacaine (Fig. 1a and b). The cleavage of caspase-3 is essential for its activation, and cleaved caspase-3 is the pivotal executor for apoptosis initiation. We found caspase-3 activity was markedly increased after Bupivacaine exposure (Fig. 1c), suggested that Bupivacaine treatment induced the apoptosis of gastric cancer cells. The apoptotic gastric cancer cells in early stage and late stage were counted through flow cytometry. As shown in Fig. 1d, the apoptosis rate was notably up-regulated in Bupivacaine treatment group compared with Control group. The number of migration and invasion cells was decreased after Bupivacaine treatment (Fig. 1e and f), suggested that Bupivacaine suppressed the metastasis of gastric cancer cells. Overall, Bupivacaine impeded the progression of gastric cancer in vitro.

### Bupivacaine restrains the aerobic glycolysis of gastric cancer cells

**Fig. 2:**
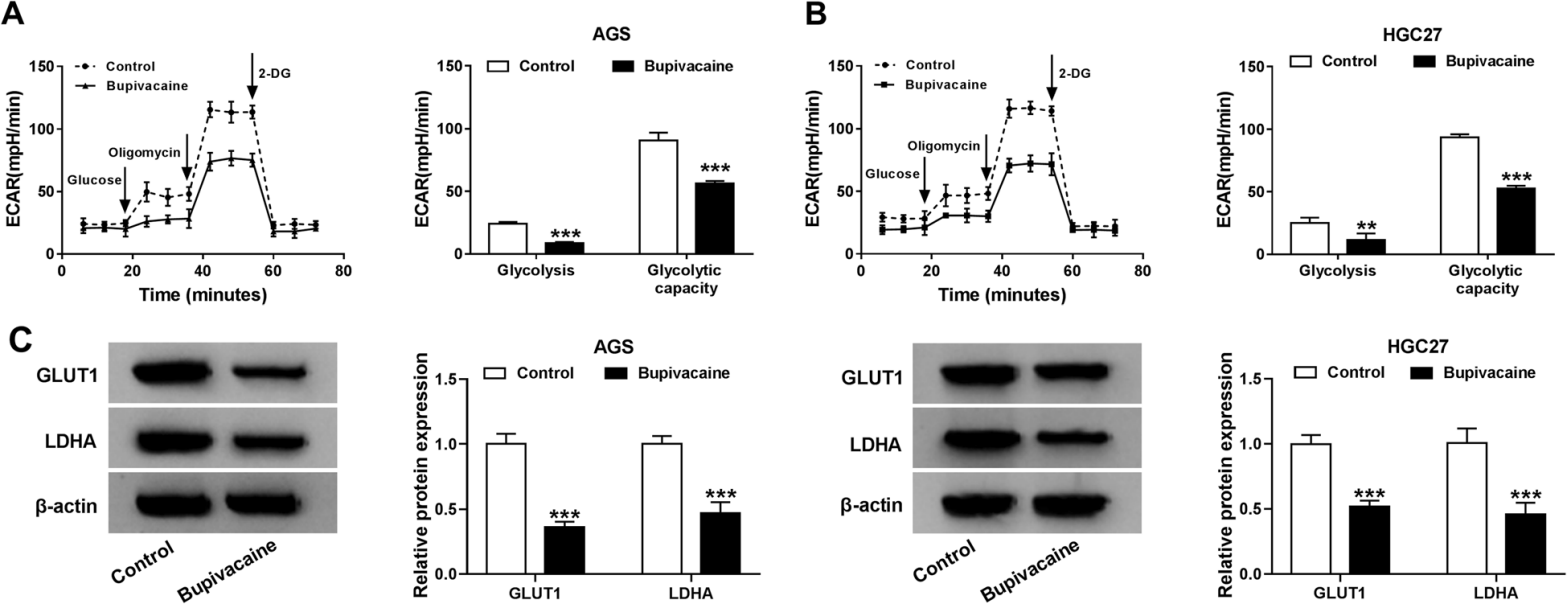
Bupivacaine restrains the aerobic glycolysis of gastric cancer cells. **a-c** AGS and HGC27 cells were exposed to 10 μg/mL Bupivacaine for 48 h. **a** and **b** Glucose, Oligomycin and 2-DG were sequentially injected. The ECAR was analyzed using the Seahorse XF 96 Extracellular Flux Analyzer to identify the glycolysis and glycolytic capacity. **c** Western blot assay was conducted to detect the expression of GLUT1 and LDHA in AGS and HGC27 cells treated with 10 μg/mL Bupivacaine for 48 h or not. **P < 0.01, ***P < 0.001

Aerobic glycolysis is an important hallmark of cancers. We tested the influence of Bupivacaine on the glycolytic metabolism of gastric cancer cells through detecting the ECAR and glycolysis-associated proteins. Glycolysis and glycolytic capacity were ascertained via ECAR values. After Bupivacaine treatment, the glycolysis and glycolytic capacity were both reduced compared with Control group (Fig. 2a and b), suggested that Bupivacaine exposure inhibited the glycolysis of gastric cancer cells. Furthermore, the protein expression of GLUT1 and LDHA was both down-regulated in Bupivacaine treatment group in comparison with that in Control group (Fig. 2c). Taken together, Bupivacaine treatment suppressed the glycolysis of gastric cancer cells.

### Circ_0000376 and miR-145-5p levels are regulated by bupivacaine in gastric cancer cells

**Fig. 3:**
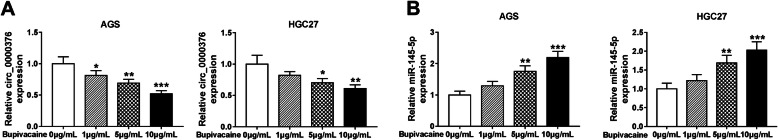
Circ_0000376 and miR-145-5p levels are regulated by Bupivacaine in gastric cancer cells. **a** and **b** AGS and HGC27 cells were stimulated with different concentrations of Bupivacaine (1 μg/mL, 5 μg/mL or 10 μg/mL) for 48 h. qRT-PCR was implemented to examine the expression of circ_0000376 and miR-145-5p in these gastric cancer cells. *P < 0.05, **P < 0.01, ***P < 0.001

To explore the mechanism involved in Bupivacaine-mediated influences in gastric cancer cells, the crucial molecules were sought. Circ_0000376 level was decreased with Bupivacaine treatment in gastric cancer cells in a dose-dependent manner (Fig. 3a). Furthermore, Bupivacaine exposure up-regulated the expression of miR-145-5p, especially in high concentration Bupivacaine group (Fig. 3b). The abnormal expression of circ_0000376 and miR-145-5p after Bupivacaine treatment might imply their important roles.

### Circ_0000376 enhances the malignant behaviors of gastric cancer cells in vitro

**Fig. 4:**
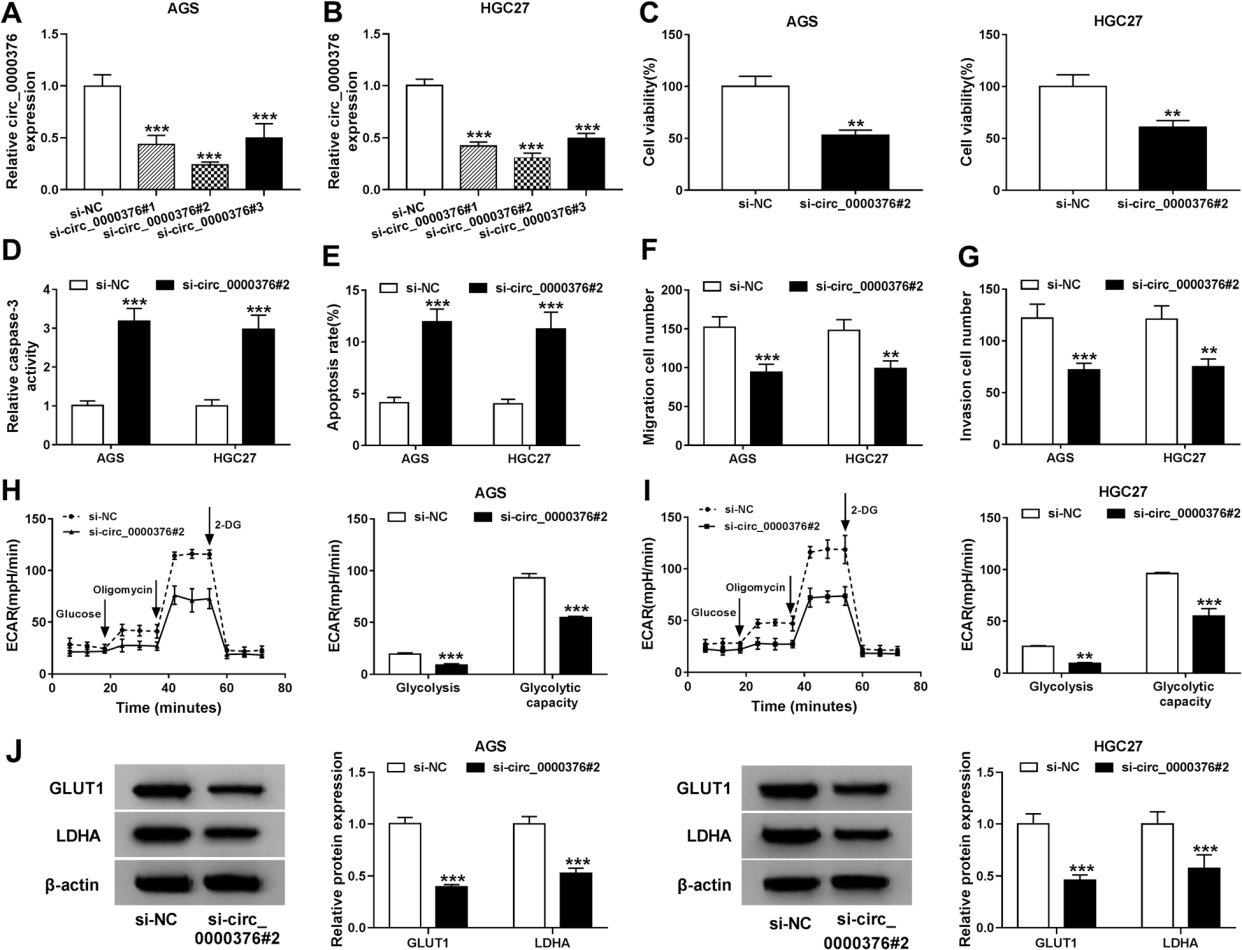
Circ_0000376 enhances the malignant behaviors of gastric cancer cells in vitro. **a** and **b** AGS and HGC27 cells were transfected with si-NC, si-circ_0000376#1, si-circ_0000376#2 or si-circ_0000376#3, respectively. Circ_0000376 abundance in these transfected gastric cancer cells was examined by qRT-PCR. **c-j** AGS and HGC27 cells were transfected with si-NC or si-circ_0000376#2, respectively. **c** CCK8 assay was utilized to assess the survival of gastric cancer cells. **d** and **e** The apoptosis of gastric cancer cells was measured through detecting the caspase-3 activity and apoptosis rate using Caspase-3 activity detection kit and flow cytometry, respectively. **f** Migration ability in si-NC or si-circ_0000376#2 transfected gastric cancer cells was evaluated by transwell migration assay. **g** Transwell invasion assay was conducted to analyze the invasion ability of gastric cancer cells. **h** and **i** The ECAR values of glycolysis and glycolytic capacity were analyzed by the Seahorse XF 96 Extracellular Flux Analyzer. **j** The protein levels of GLUT1 and LDHA in transfected gastric cancer cells were measured by Western blot assay. **P < 0.01, ***P < 0.001

Small interfering RNAs against circ_0000376 were designed to specifically silence circ_0000376 in gastric cancer cells. Among these three siRNAs targeting circ_0000376, si-circ_0000376#2 possessed higher transfection efficiency than si-circ_0000376#1 and circ_0000376#3 (Fig. 4a and b), thus we selected circ_0000376#2 for loss-of-function experiments. Circ_0000376 silencing resulted in decreased cell viability (Fig. 4c). Furthermore, significant up-regulation in caspase-3 activity and apoptosis rate was observed in si-circ_0000376#2 transfected gastric cancer cells compared with that in si-NC group (Fig. 4d and e). The results of transwell migration and invasion assays indicated that circ_0000376 knockdown notably impeded the migration and invasion capacities of gastric cancer cells (Fig. 4f and g). We also explored if circ_0000376 silencing restrained the glycolysis of gastric cancer cells. The glycolysis and glycolytic capacity were significantly decreased when circ_0000376 was silenced compared with si-NC group (Fig. 4h and i). There was an obvious down-regulation in the expression of GLUT1 and LDHA in si-circ_0000376#2 transfected gastric cancer cells rather than si-NC group (Fig. 4j). These findings demonstrated that circ_0000376 promoted the progression of gastric cancer through accelerating the viability, metastasis and glycolysis and suppressing the apoptosis of gastric cancer cells.

### Circ_0000376 acts as a sponge of miR-145-5p in gastric cancer cells

**Fig. 5:**
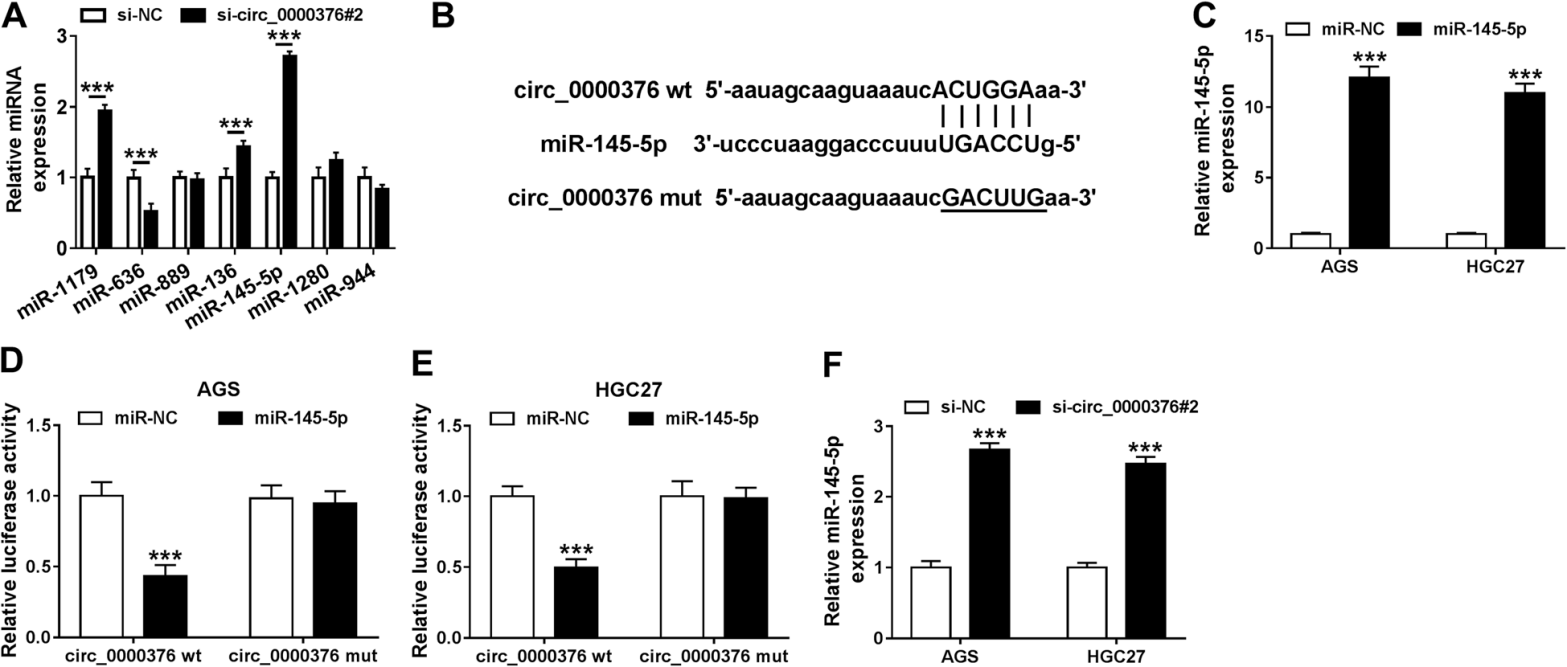
Circ_0000376 acts as a sponge of miR-145-5p in gastric cancer cells. **a** Circular RNA Interactome database was used to find the interacted-miRNAs of circ_0000376. The expression of these seven candidate miRNAs was measured in AGS cells transfected with si-NC or si-circ_0000376#2 via qRT-PCR. **b** The schematic represented the binding sites between circ_0000376 and miR-145-5p. The mutant binding sites with miR-145-5p in circ_0000376 were also shown as circ_0000376 mut. **c** The transfection efficiency of miR-145-5p in gastric cancer cells was evaluated by qRT-PCR. **d** and **e** Dual-luciferase reporter assay was conducted to analyze the direct binding between miR-145-5p and circ_0000376. AGS and HGC27 cells were co-transfected with miR-NC or miR-145-5p and circ_0000376 wt or circ_0000376 mut. The luciferase activities in these four different groups in AGS and HGC27 cells were detected. **f** MiR-145-5p level in si-NC or si-circ_0000376#2 transfected gastric cancer cells was measured by qRT-PCR. ***P < 0.001

We explored circ_0000376 specific miRNA targets by Circular RNA Interactome database. Among seven candidate miRNA targets of circ_0000376, miR-145-5p was the most up-regulated miRNA with the silencing of circ_0000376 in AGS cells (Fig. 5a), thereby we further explored the interaction between circ_0000376 and miR-145-5p. The binding sequence between circ_0000376 and miR-145-5p predicted by Circular RNA Interactome database was shown in Fig. 5b. MiR-145-5p mimic transfection significantly up-regulated miR-145-5p level in AGS and HGC27 cells (Fig. 5c). We co-transfected miR-NC or miR-145-5p and circ_0000376 wt or circ_0000376 mut into AGS and HGC27 cells. MiR-145-5p transfection notably decreased the luciferase activity in circ_0000376 wt group compared with miR-NC and circ_0000376 wt group, while miR-145-5p had no effect on the luciferase activity in circ_0000376 mut group compared with miR-NC and circ_0000376 mut group (Fig. 5d and e), suggesting the direct combination between miR-145-5p and circ_0000376 in gastric cancer cells. To test whether miR-145-5p level was regulated by circ_0000376 through the direct interaction, si-circ_0000376#2 was transfected into AGS and HGC27 cells. As shown in Fig. 5f, miR-145-5p was remarkably up-regulated with the silencing of circ_0000376 in gastric cancer cells. Collectively, circ_0000376 down-regulated miR-145-5p through the direct interaction in gastric cancer cells.

### Bupivacaine-induced influences in gastric cancer cells are largely reversed by the introduction of circ_0000376 overexpression plasmid or anti-miR-145-5p

**Fig. 6:**
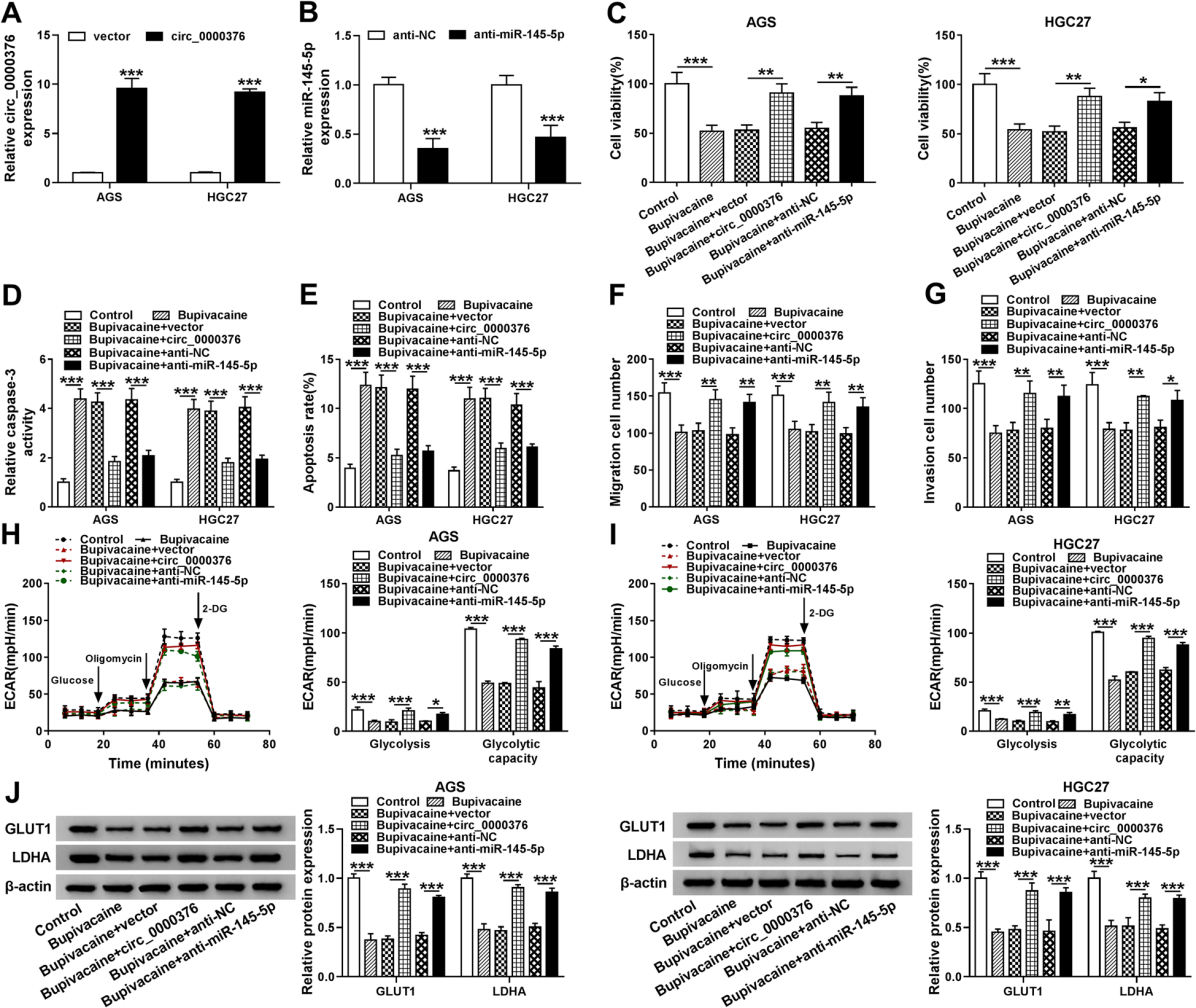
Bupivacaine-induced influences in gastric cancer cells are largely reversed by the introduction of circ_0000376 overexpression plasmid or anti-miR-145-5p. **a** and **b** The transfection efficiencies of circ_0000376 and anti-miR-145-5p in gastric cancer cells were assessed by qRT-PCR. **c**-**j** We treated AGS and HGC27 cells with Bupivacaine (10 μg/mL, 48 h), Bupivacaine + vector, Bupivacaine + circ_0000376, Bupivacaine + anti-NC or Bupivacaine + anti-miR-145-5p. **c** Cell viability in different groups was detected by CCK8 assay. **d** Caspase-3 activity was measured using Caspase-3 activity detection kit. **e** The apoptosis rate was analyzed by flow cytometry. **f** and **g** Transwell migration and invasion assays were implemented to analyze the migration and invasion abilities of gastric cancer cells. **h** and **i** The ECAR was analyzed by the Seahorse XF 96 Extracellular Flux Analyzer to assess the glycolytic metabolism of gastric cancer cells. **j** The expression of GLUT1 and LDHA in AGS and HGC27 cells was detected by Western blot assay. *P < 0.05, **P < 0.01, ***P < 0.001

On the basis of the regulatory relationship and functional association between Bupivacaine and circ_0000376 or miR-145-5p, we proposed the hypothesis that Bupivacaine functioned through regulating circ_0000376/miR-145-5p axis. To verify our hypothesis, AGS and HGC27 cells were divided into six groups as followed: Control, Bupivacaine, Bupivacaine + vector, Bupivacaine + circ_0000376, Bupivacaine + anti-NC and Bupivacaine + anti-miR-145-5p. Prior to functional experiments, the overexpression and knockdown efficiencies of circ_0000376 and anti-miR-145-5p in gastric cancer cells were assessed by qRT-PCR. As shown in Fig. 6a, circ_0000376 level was notably elevated in AGS and HGC27 cells transfected with circ_0000376 overexpression plasmid compared with vector group. As mentioned in Fig. 6b, anti-miR-145-5p transfection significantly down-regulated miR-145-5p level in gastric cancer cells than that in anti-NC group. Bupivacaine-induced suppression on the viability and promotion on the apoptosis of gastric cancer cells were largely attenuated by the overexpression of circ_0000376 or the silencing of miR-145-5p (Fig. 6c-e). The migration and invasion abilities of gastric cancer cells were recovered with the addition of circ_0000376 or anti-miR-145-5p which were suppressed by Bupivacaine treatment (Fig. 6f and g). Furthermore, Bupivacaine-mediated suppression on the ECAR of gastric cancer cells was largely counteracted in Bupivacaine + circ_0000376 co-transfected group and Bupivacaine + anti-miR-145-5p co-transfected group (Fig. 6h and i), suggested that Bupivacaine suppressed the glycolytic metabolism of gastric cancer cells through down-regulating circ_0000376 and up-regulating miR-145-5p. The abundance of GLUT1 and LDHA protein was reduced with Bupivacaine treatment, while the overexpression of circ_0000376 or the interference of miR-145-5p further regained the protein levels of GLUT1 and LDHA in AGS and HGC27 cells (Fig. 6j). Taken together, Bupivacaine suppressed the malignant potential of gastric cancer cells through modulating circ_0000376/miR-145-5p axis.

## Discussion

Local anesthetic used during the operation benefits cancer patients with breast cancer, common cancer and gastric cancer through suppressing the metastasis and recurrence of tumors [22, 23]. Bupivacaine is one of the amide-linked local anesthetics. Amide-linked local anesthetics have been identified to directly restrain the growth, viability and motility of tumor cells [24, 25]. Dan et al. reported that Bupivacaine suppressed the progression of gastric cancer [26]. However, the precise molecular mechanisms behind Bupivacaine in the progression of gastric cancer remain to be revealed. We aimed to seek the crucial molecules that were implicated in the working mechanism of Bupivacaine. Our results suggested that Bupivacaine impeded the progression of gastric cancer through down-regulating circ_0000376, thus elevating the level of miR-145-5p.

Bupivacaine dose-dependently suppressed the viability of gastric cancer cells. Furthermore, Bupivacaine at the concentration of 10 μg/mL notably induced the apoptosis while impaired the metastasis and aerobic glycolysis of gastric cancer cells. The anti-tumor role of Bupivacaine was consistent with the previous work [26]. Circ_0000376 was found to be significantly down-regulated with the treatment of Bupivacaine in gastric cancer cells. Anesthetics have been reported to inhibit the progression of multiple cancers through regulating ncRNAs. For example, Propofol impeded the proliferation and motility of gastric cancer cells through targeting miR-29/MMP2 signaling [27]. Sevoflurane restrained the metastasis of glioma cells through modulating miR-146b-5p/MMP16 axis [28]. Zhang et al. found that Bupivacaine induced the apoptosis and neurotoxicity of neuroblastoma cells through regulating miR-132/IGF1R axis [29]. As for circ_0000376, Jiang et al. found that circ_0000376 was abnormally up-regulated in gastric cancer [16]. We wondered if Bupivacaine suppressed the progression of gastric cancer through down-regulating circ_0000376. In the current study, through conducting loss-of-function experiments, we found that circ_0000376 accelerated the viability, migration, invasion and glycolytic metabolism and impeded the apoptosis of gastric cancer cells. Through treating gastric cancer cells with Bupivacaine or co-treating with Bupivacaine and circ_0000376 ectopic expression plasmid, we found Bupivacaine suppressed the malignant behaviors of gastric cancer cells through down-regulating circ_0000376.

MiR-145-5p was identified as a tumor suppressor in a variety of cancers. For instance, Wei et al. claimed that PVT1 facilitated the proliferation, migration and invasion while suppressed the apoptosis of non-small cell lung cancer cells through sponging miR-145-5p to up-regulate ITGB8 [30]. Chen et al. found that miR-145-5p impeded the proliferation and motility of colorectal cancer cells through targeting CDCA3 [31]. Wang et al. found that miR-145-5p acted as a direct target of KCNQ1OT1 to suppress the progression of bladder cancer through suppressing PCBP2 [32]. CircDUSP16 contributed to the development of gastric cancer through sponging miR-145-5p [20]. Furthermore, miRNAs exerted crucial roles in the working mechanisms of anaesthetics in cancers [33–35]. Among these reports, lidocaine, as one of the amide-linked local anaesthetics, was found to suppress the growth and metastasis of gastric cancer cells through elevating the level of miR-145 [33]. In this study, miR-145-5p was verified as a direct target of circ_0000376 in gastric cancer cells. To test whether Bupivacaine functioned through regulating the level of miR-145-5p, we co-treated gastric cancer cells with Bupivacaine and anti-miR-145-5p. Rescue experiments showed that Bupivacaine restrained the progression of gastric cancer through up-regulating miR-145-5p.

In summary, local anesthetic Bupivacaine suppressed the viability, migration, invasion and glycolytic metabolism while induced the apoptosis of gastric cancer cells. Furthermore, the direct interaction between circ_0000376 and miR-145-5p was firstly identified in this study, and Bupivacaine was found to restrain the progression of gastric cancer through decreasing circ_0000376 level, thus up-regulating the level of miR-145-5p.

## Supplementary information

**Additional file 1: Figure S1.** The images of flow cytometry and transwell assays in Fig. 6. (A) Cell population in four quadrants in different treatment groups of Fig. 6e was shown. The apoptosis rate indicated the percentage of GC cells with FITC^+^ and PI^+/−^. (B) Representative images of transwell migration assay of Fig. 6f were shown. (C) Representative images of transwell invasion assay of Fig. 6g were displayed.

**Additional file 2.** Western blots.

## Data Availability

The datasets used and/or analyzed during the current study are available from the corresponding author on reasonable request.

## Abbreviations

CCK8: Cell counting kit-8
ECAR: Extracellular acidification rate
GLUT1: Glucose transporter type 1
LDHA: Lactic dehydrogenase A
qRT-PCR: Quantitative real-time polymerase chain reaction
circ_0000376: Circular RNA 0000376
miR-145-5p: microRNA-145-5p
