# Supplementary figures and images for: Bupivacaine suppresses the progression of gastric cancer through regulating circ_0000376/miR-145-5p axis
Source: BMC Anesthesiol. 2020 Oct 30;20:275. doi: 10.1186/s12871-020-01179-4 (PMC7597012; doi:10.1186/s12871-020-01179-4)

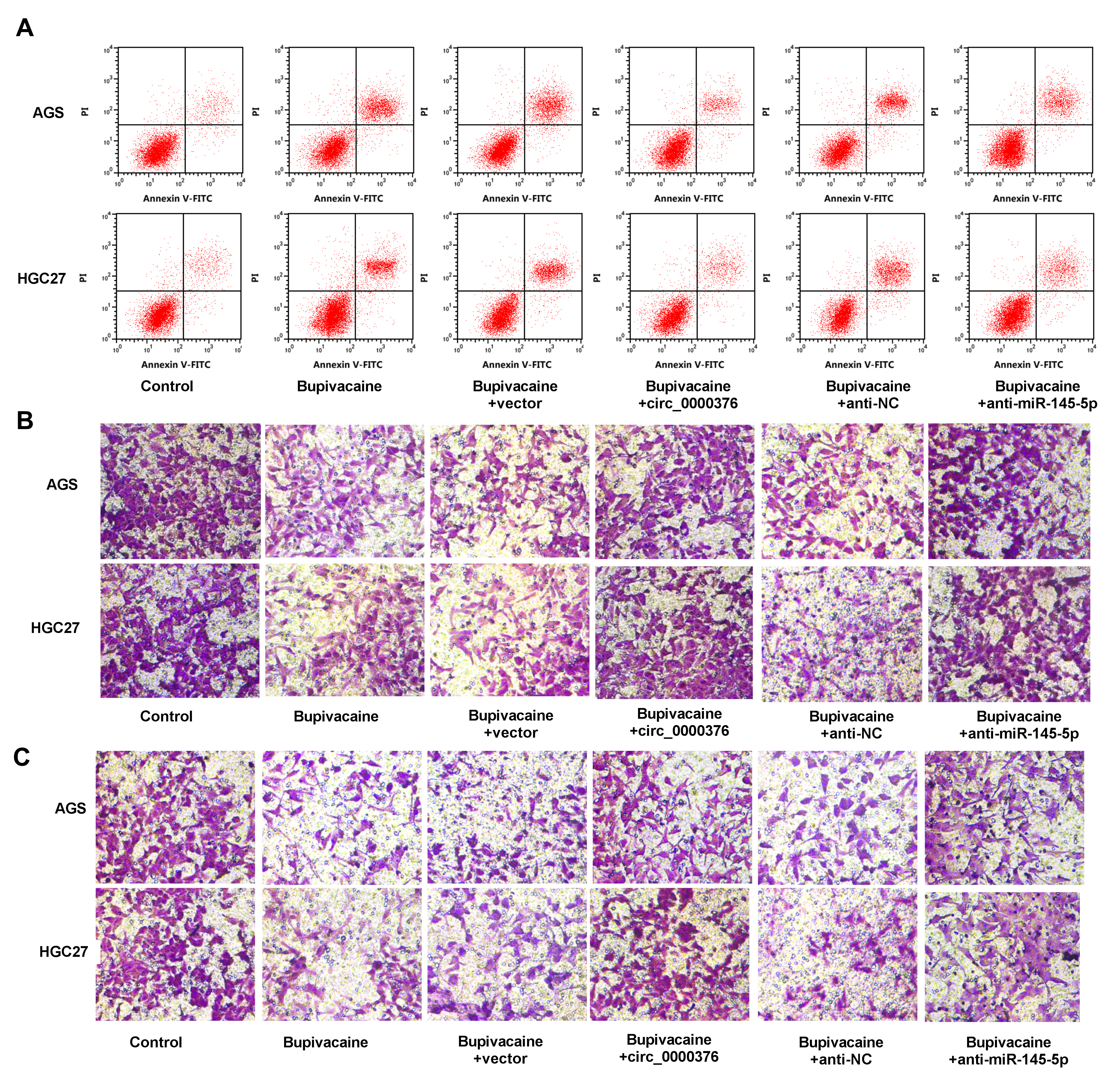

Supplement: Supplementary file 1 — Additional file 1: Figure S1. The images of flow cytometry and transwell assays in Fig. 6. (A) Cell population in four quadrants in different treatment groups of Fig. 6e was shown. The apoptosis rate indicated the percentage of GC cells with FITC+ and PI+/−. (B) Representative images of transwell migration assay of Fig. 6f were shown. (C) Representative images of transwell invasion assay of Fig. 6g were displayed. [file 12871_2020_1179_MOESM1_ESM.tif]

Fig 2C  
AGS

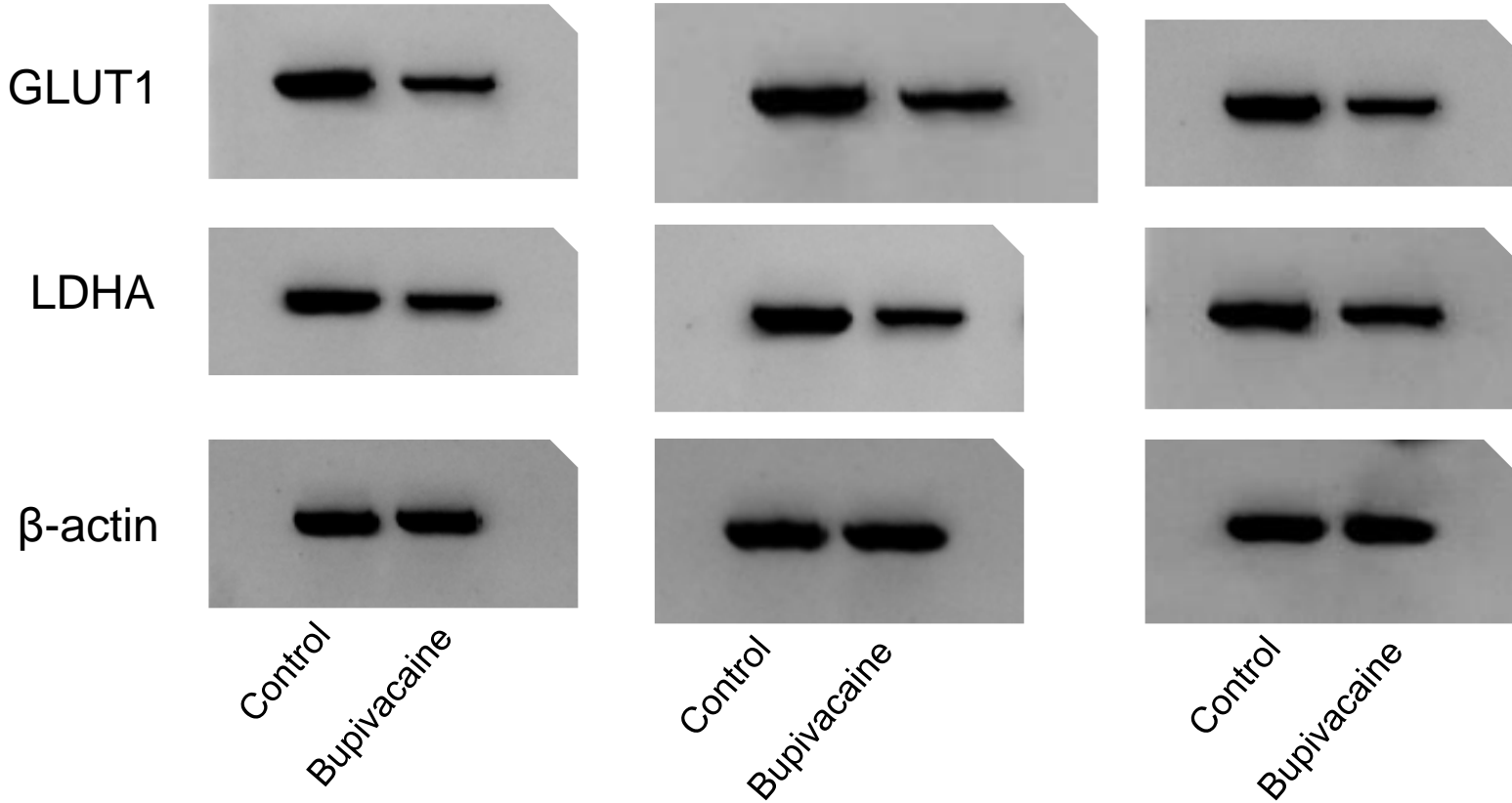

Fig 2C  
HGC27

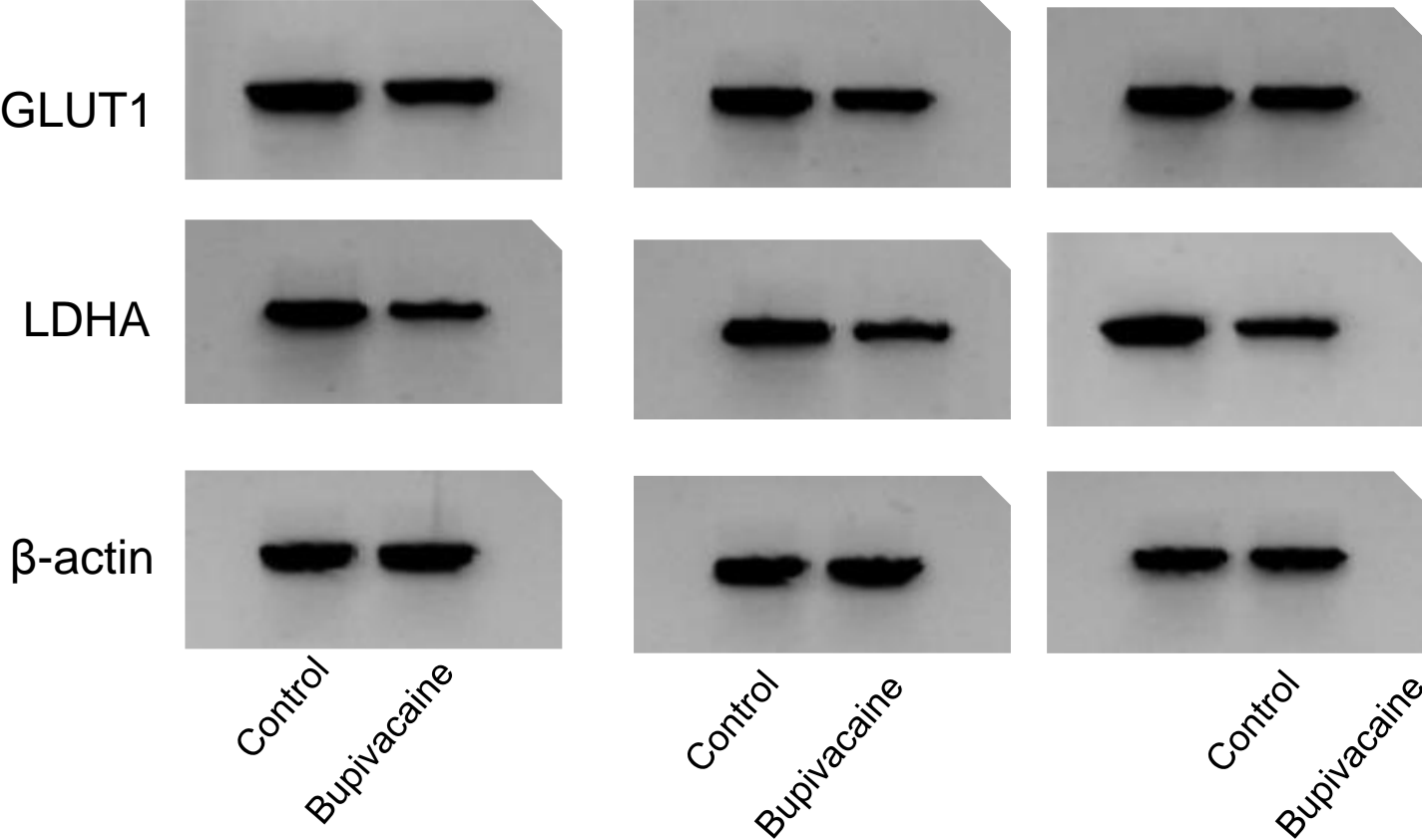

Fig 4J  
AGS

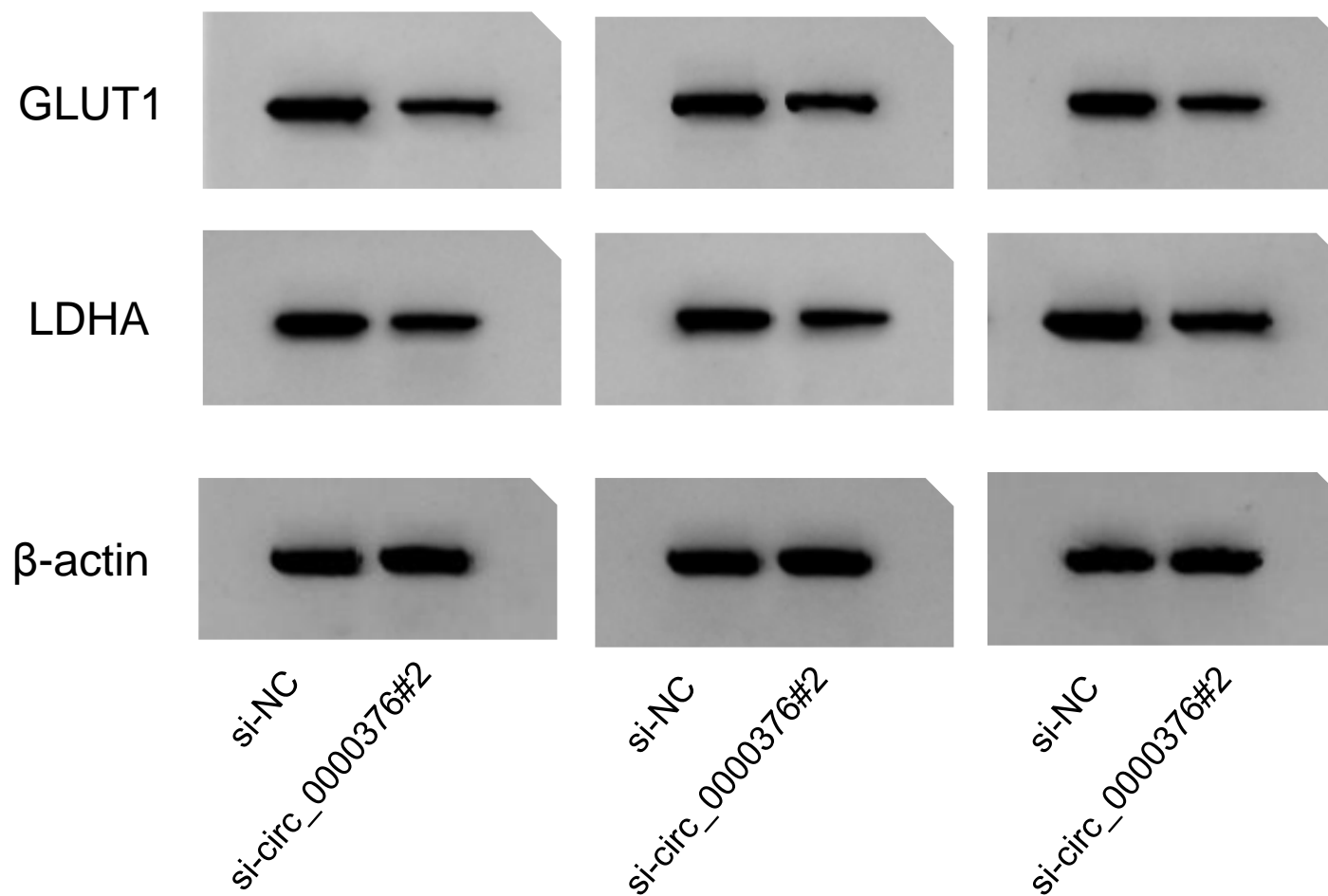

Fig 4J  
HGC27

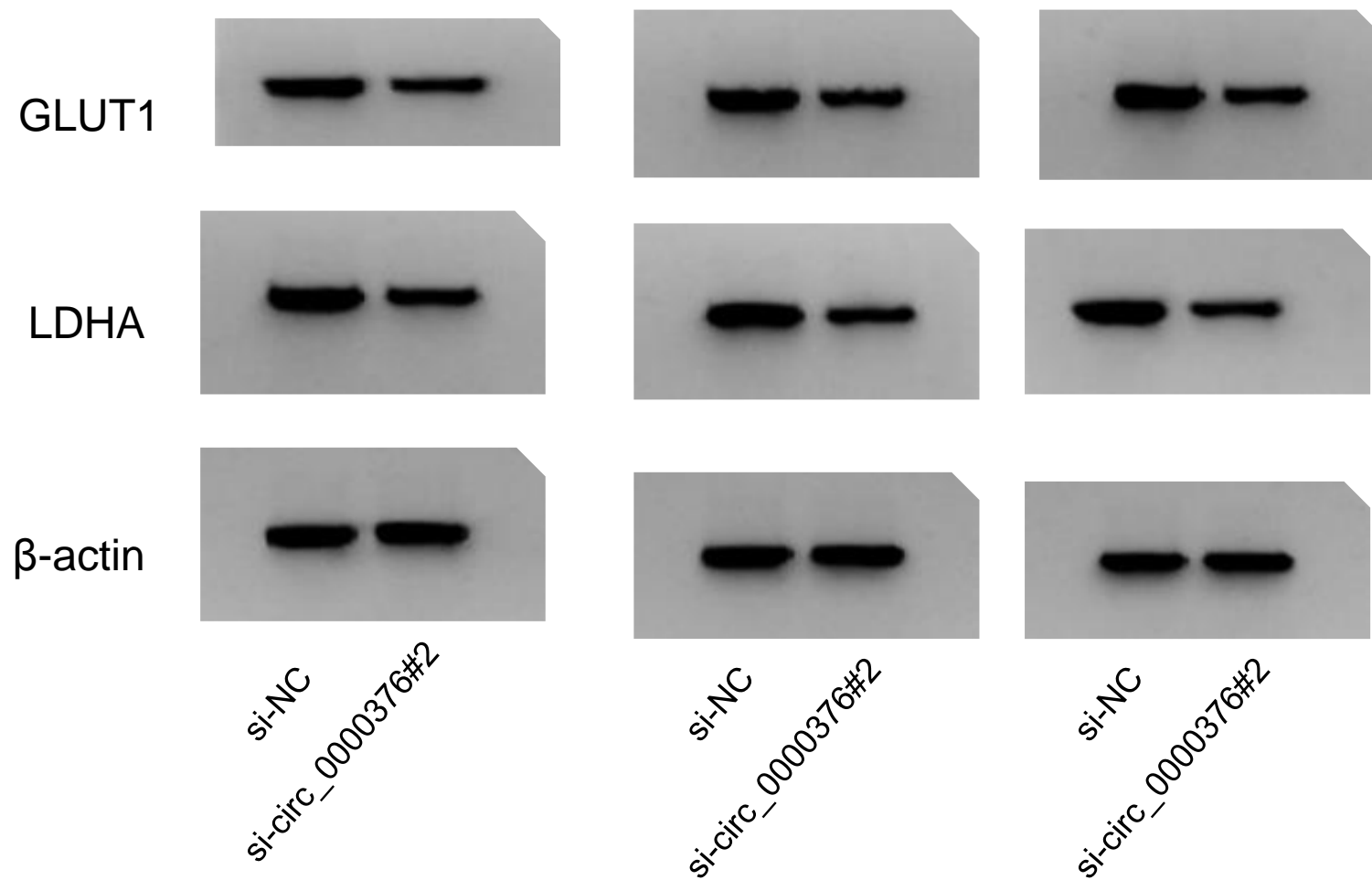

Fig 6J  
AGS

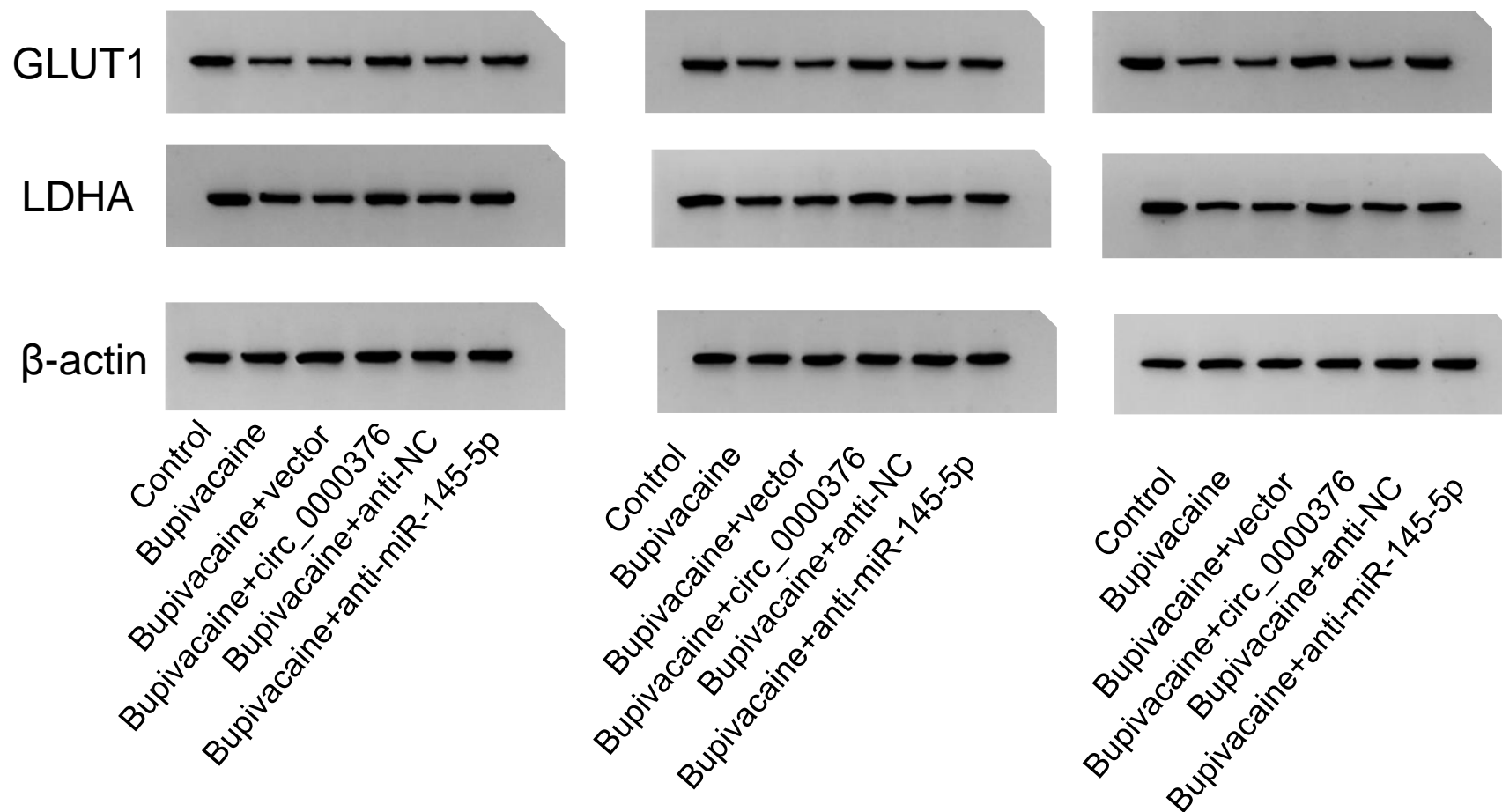

Fig 6J  
HGC27

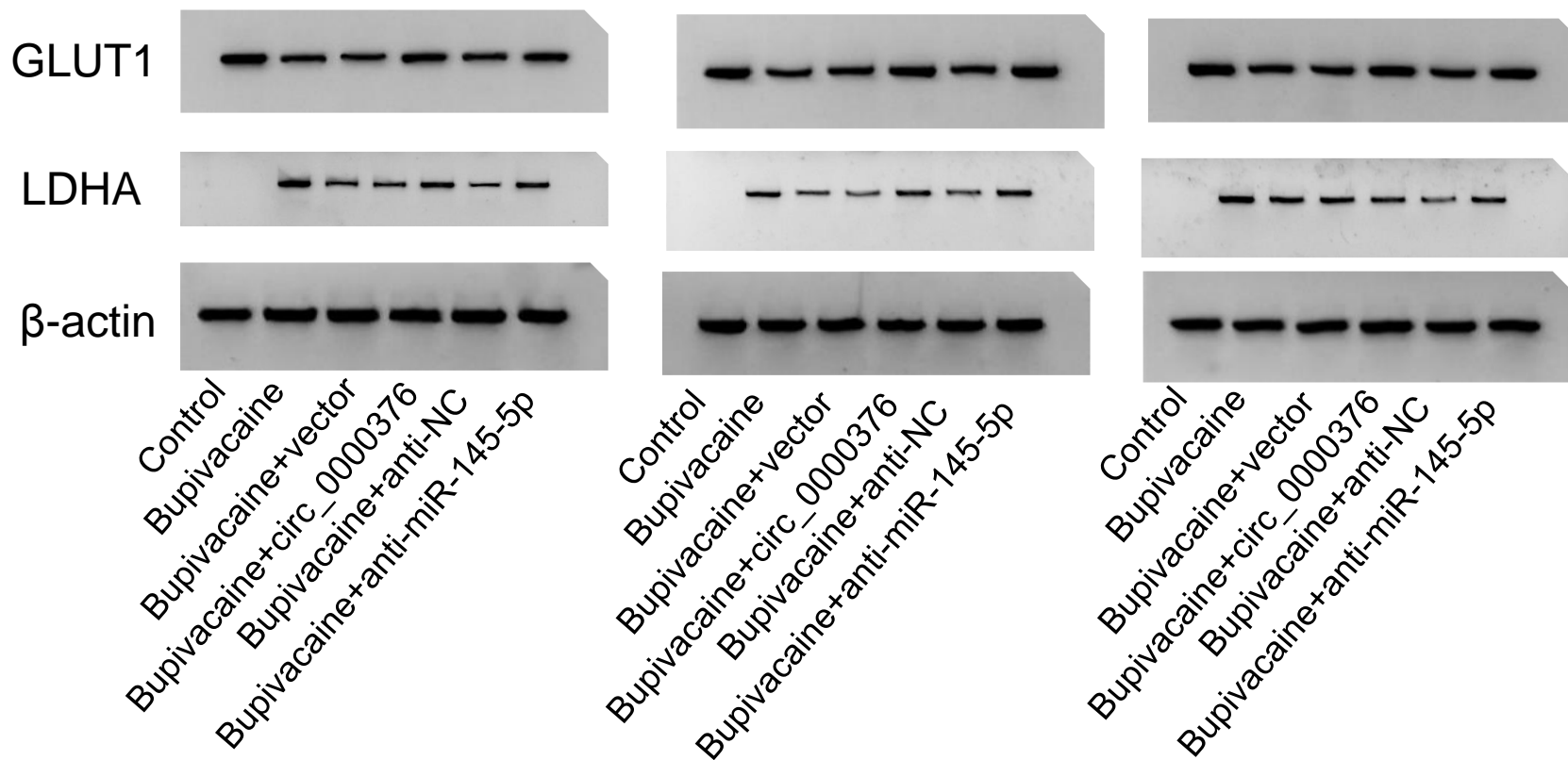

Supplement: Supplementary file 2 — Additional file 2. Western blots. [file 12871_2020_1179_MOESM2_ESM.pdf]
